# Delayed Lead Perforation: Can We Ever Let the Guard Down?

**DOI:** 10.4061/2010/741751

**Published:** 2010-07-25

**Authors:** Venkata M. Alla, Yeruva M. Reddy, William Abide, Tom Hee, Claire Hunter

**Affiliations:** ^1^Division of Cardiology, Creighton University Medical Center, Omaha, NE 68131, USA; ^2^Department of Internal Medicine, Creighton University Medical Center, Omaha, NE 68131, USA

## Abstract

Lead perforation is a major complication of cardiac rhythm management devices (CRMD), occurring in about 1%. While most lead perforations occur early, numerous instances of delayed lead perforation (occurring >30 days after implantation) have been reported in the last few years. Only about 40 such cases have been published, with the majority occurring <1 year after implantation. Herein, we describe the case of an 84-year-old female who presented with recurrent syncope and was diagnosed to have delayed pacemaker lead perforation 4.8 years after implantation. Through this report, we intend to highlight the increasing use of CRMD in elderly patients, and the lifelong risk of complications with these devices. Presentation can be atypical and a high index of suspicion is necessary for diagnosis.

## 1. Introduction

Increasing burden of cardiovascular disease and broadening indications have led to a significant increase in the implantation of cardiac rhythm management devices (CRMDs) [1–3]. Approximately 70% of CRMD recipients are ≥65 years of age and 20%–35% are older than 80 years [1–4]. Cardiac perforation is a major complication of CRMD implantation and can be acute, subacute, or delayed. Herein, we describe a case of delayed pacemaker lead perforation (DLP) occurring 4.8 years after implantation. Through this report, we emphasize the need to consider device related complications in the differential diagnoses of patients with CRMD, even years after implantation.

## 2. Case Summary

**Figure 1:**
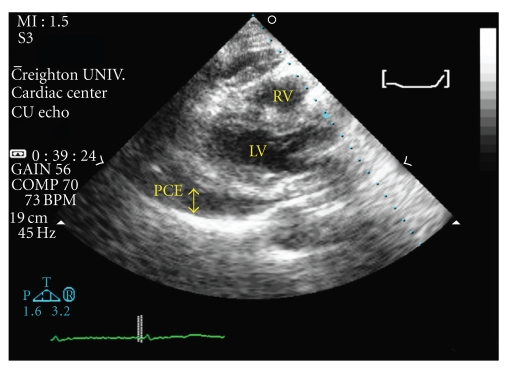
Transthoracic echocardiogram in parasternal long axis view showing the large pericardial effusion. LV: left ventricle; RV: right ventricle; PCE: pericardial effusion.

An 84-year-old female presented with two episodes of transient loss of consciousness over 24 hours. She was initially evaluated in the emergency room of an outlying hospital where physical examination, electrocardiogram (ECG), and chest X-ray were unremarkable. Subsequently, she was transferred to our hospital for further evaluation. She denied chest pain, palpitations, dyspnea, or orthostatic dizziness. She was seated at the time of the index events, had no premonitory symptoms, and regained consciousness within a few minutes. Past medical history was significant for rheumatoid arthritis, paroxysmal atrial fibrillation, and symptomatic sinus pauses requiring a dual chamber pacemaker placement. The pacemaker (Medtronic, KDR901 Kappa DR; leads: Medtronic 5076 CapSure Fix Novus) was implanted in December, 2004 with the right ventricular lead in the apex and right atrial lead in the appendage. Her medications included warfarin, metoprolol, prednisone, and amiodarone. Pulse rate was 65/minute, blood pressure was 96/58 mmHg, and physical examination was significant for prominent jugular venous pulsations, and distant heart sounds. Metabolic panel and blood counts were normal and INR was therapeutic at 2.1. ECG revealed normal sinus rhythm without any abnormalities, chest X-ray demonstrated mild cardiomegaly with stable atrial/ventricular lead position and computed tomography scan of the head was unremarkable. Transthoracic echocardiogram revealed a large pericardial effusion with early echocardiographic signs of cardiac tamponade (Figure 1). Pacemaker evaluation revealed normal function; lead impedance, sensing and pacing thresholds were stable compared to evaluation 3 months prior. Previous echocardiogram, also done 3 months prior was unremarkable except for mild left ventricular hypertrophy. She underwent emergent surgical pericardiocentesis and about 600 mL of bloody fluid was drained. During the surgery, a defect in the right ventricular myocardium was visualized and repaired with sutures. The ventricular lead was in close proximity but there was no definite protrusion of the tip through the defect. A pericardial window was created and the right ventricular lead was successfully repositioned under transesophageal echo guidance. Pericardial fluid cultures were sterile, cytology was negative for malignant cells, and pericardial biopsy was normal. Further hospital stay was uneventful and she was discharged on her home medication regimen. She has had no recurrent symptoms or pericardial effusion at 1 year followup.

## 3. Discussion

The frequency of lead perforation varies between 0.1%–0.8% for pacemaker leads and 0.6%–5.2% for defibrillators [5–7]. Lead perforation is considered to be acute when it occurs within 5–7 days after implantation; subacute when it occurs between 7–30 days; delayed when it occurs beyond 30 days after implantation. While most lead perforations occur early, DLP has been increasingly reported in the last few years. We identified a total of 38 patients with DLP in our review of literature (Table 1). The vast majority of these occurred between 6 weeks and 1 year from implantation; 7/38 patients had DLP beyond one year and only one patient had this complication >3 years after implantation. Our patient presented close to 5 years after implantation, which is unusually long and underscores the potential lifelong risk. 

 Though the lead was not visualized in the pericardial space we believe that DLP is the most likely explanation in our patient. The lead was noted to be in close proximity to the defect in the RV myocardium, effusion was bloody, and there was no alternative etiology for myocardial perforation or pericardial effusion. However, the exact timing of lead perforation is unclear. Though chronic perforation cannot definitively be excluded, a normal echocardiogram 3 months prior suggests that this was more recent. Presentation of DLP is variable ranging from minimal or no symptoms to cardiac tamponade and sudden death. Several known risk factors for acute lead perforation exist; these include patient characteristics like advanced age, female gender; hardware factors like defibrillator versus pacemaker leads, smaller lead diameter, increased lead stiffness, active versus passive.

fixation tips; and miscellaneous factors like use of steroids in the perioperative period [8]. However, the role of these factors in DLP has not been clearly established.

 In summary, patients continue to be at risk for potentially grave complications like lead perforation many years after implantation of CRMD. Symptoms can be subtle or atypical especially in those with advanced age and a high index of suspicion is necessary for timely diagnosis.

## Figures and Tables

**Table 1:** Previously published cases of delayed lead perforation showing patient age and the delay from implantation to perforation.

Author	Publication	Number of patients	Age in years	Time from implantation to perforation	Comments
Ahmet Akyol	Pacing Clin Electrophysiol 2005; 28:350–351	1	24	6 months	
Khan MN	Pacing Clin Electrophysiol 2005; 28:251–253	3	26, 71, 81	6–10 months	
Velavan P	Heart 2003; 89:364	1	64	1 month	
Satpathy R	Pacing Clin Electrophysiol 2008; 31:10–12	1	72	10 months	
Haq SA	Angiology 2008; 59:619	1	86	16 months	
Ellenbogen KA	Pacing Clin Electrophysiol 2002; 25:1155–1158	3	73, 72, 42	30 days	3/5 cases reported here were late perforations others being sub acute.
Kautzner J	Pacing Clin Electrophysiol 2001; 24:116–118	1	36	23 months	Symptoms of pericarditis without perforation 4 months after implantation.
Polin GM	Am J Cardiol. 2006 15; 98:223–5	5	38, 55, 79, 85, 88	6 weeks–3years	
Laborderie J	Am J Cardiol 2008; 102:1352–1355	8	35, 50, 40, 53, 64, 78, 81, 84	1–3.5 months	8/11 cases reported here were late perforations.
Fisher JD	Pacing Clin Electrophysiol 2008; 31:7–9	1	71	38 days	
Krivan L	Pacing Clin Electrophysiol 2008; 31:3–6	1	47	1 month	1/2 cases reported here was late perforation the other was acute.
Lloyd MS	Pacing Clin Electrophysiol 2008; 31:784–785	1	68	6 weeks	1/3 cases reported here was late perforation others being sub acute.
Suri R	Heart Rhythm. 2007 Sep; 4(9):1248–9	1	NA	6 weeks	1/5 cases reported here was late perforation others being sub acute.
Sadamatsu K	J Cardiol. 2009; 53(1):150–3.	1	NA	9 months	asymptomatic
Kanoh M	Kyobu Geka. 1994; 47:730–1	1	80	3 months	asymptomatic
Park RE	Pacing Clin Electrophysiol 2008; 31:785–786	1	72	6 weeks	
Singhal S	Circulation. 2007; 115:e391-2	1	50	7 years	Perforated through the rib.
Wiegand UK	Pacing Clin Electrophysiol. 2003; 26(10):1961-9	1	NA	3 years	1 case of delayed perforation in 116 implanted leads.
Sanoussi A	Pacing Clin Electrophysiol. 2005; 28:723–725	1	79	1 month	
Tziakas D	Europace 2009; 11:968–969.	1	84	5 weeks	
Celik T	Europace 2009; 11:963–965.	2	73, 65	8 months, 2 years	
Tavernier R	Europace. 2009; 11:966–7	1	74	1 month	
